# Bat-mouse bone marrow chimera: a novel animal model for dissecting the uniqueness of the bat immune system

**DOI:** 10.1038/s41598-018-22899-1

**Published:** 2018-03-16

**Authors:** Kylie Su Mei Yong, Justin Han Jia Ng, Zhisheng Her, Ying Ying Hey, Sue Yee Tan, Wilson Wei Sheng Tan, Sergio Erdal Irac, Min Liu, Xue Ying Chan, Merry Gunawan, Randy Jee Hiang Foo, Dolyce Hong Wen Low, Ian Hewitt Mendenhall, Yok Teng Chionh, Charles-Antoine Dutertre, Qingfeng Chen, Lin-Fa Wang

**Affiliations:** 1 Institute of Molecular and Cell Biology, Agency for Science, Technology and Research (A*STAR), 138673 Singapore, Singapore; 2 Programme in Emerging Infectious Diseases, Duke-NUS Medical School, 169857 Singapore, Singapore; 3 Singapore Immunology Network (SIgN), Agency for Science, Technology and Research (A*STAR), 138648 Singapore, Singapore; 4 Department of Microbiology and Immunology, Yong Loo Lin School of Medicine, National University of Singapore, 119228 Singapore, Singapore; 5 Key Laboratory for Major Obstetric Diseases of Guangdong Province, The Third Affiliated Hospital of Guangzhou Medical University, Guangzhou, 510150 China

## Abstract

Bats are an important animal model with long lifespans, low incidences of tumorigenesis and an ability to asymptomatically harbour pathogens. Currently, *in vivo* studies of bats are hampered due to their low reproduction rates. To overcome this, we transplanted bat cells from bone marrow (BM) and spleen into an immunodeficient mouse strain NOD-scid IL-2R^−/−^ (NSG), and have successfully established stable, long-term reconstitution of bat immune cells in mice (bat-mice). Immune functionality of our bat-mouse model was demonstrated through generation of antigen-specific antibody response by bat cells following immunization. Post-engraftment of total bat BM cells and splenocytes, bat immune cells survived, expanded and repopulated the mouse without any observable clinical abnormalities. Utilizing bat’s remarkable immunological functions, this novel model has a potential to be transformed into a powerful platform for basic and translational research.

## Introduction

Bats are an important nidus for an extensive spectrum of viruses, ranging from Rabies, Henipavirus to SARS coronavirus (SARS-Cov), Marburg and Ebola viruses^1–7^. Being found in all continents except Antarctica, bats are not only geographically dispersed, but they also have long life spans and highly social behaviours that make them favourable hosts and vectors for disease transmission^1,8–10^. In comparison to rodents, bats have an ability to host more viruses per species^11–13^, resulting in sympatric and cross species infection between mammals^1^. Despite possessing these characteristics, bats are remarkable creatures that are highly resistant to diseases upon infection by many of the viruses they carry^10,14^. This may suggest that pathogens have a possible commensal, mutualistic relationship or specific adaptation to the bat’s immune system^9,14^. Currently, little is known about bat immune system^9^, therefore, it is of extreme importance to dissect the immune system of bats, so as to discover their seemingly unique ability in controlling infections and preventing diseases.

Multiplex biological processes often require a homogenous model for both *in vivo* and *ex vivo* analysis. The study of bat biology is limited due to reasons such as, (1) wild bats of the same genetic lineage may express a wide variation in their response to the same stimulus, (2) due to conservation and ethical reasons, species of interest cannot be captured from the wild freely and/or in large numbers^15^, (3) with innate instincts of setting up maternity colonies, it is extremely challenging to breed bats within an animal facility and their reproduction rate is much lower than rodents^16^. To date, most bat research at the cellular and molecular level has been mainly restricted to *in vitro* work using specialised bat cell lines generated in-house^17–20^. On the contrary, many research advances have been made using mice as a model for the study of various biological systems^21,22^. The mouse offers one of a kind advantage as an animal model because they are small, relatively inexpensive to maintain and most importantly, they have short generation times with an ability to produce a large number of offspring^22^. Inbred strains are almost genetically identical, and their environment can be controlled and manipulated easily^23,24^.

Over the last decade, there has been a wave of high-impact research carried out on cross-species engraftment, such as, the stable reconstitution of human immune system in immunodeficient mice (humanized mouse models)^25,26^. The development of immunodeficient mice has provided the opportunity to utilize small animal models for the study of many *in vivo* human-specific immune responses^27^. The establishment of a targeted mutation in the IL-2 receptor common gamma chain gene (IL-2Rγ^−/−^) in mice already deficient in T and B cells led to a breakthrough in the ability to engraft hematopoietic stem cells, as well as functional human lymphoid cells and tissues^28^, effectively creating human immune systems within an immunodeficient mice^24,29,30^. These humanized mice are becoming increasingly important as pre-clinical models for a range of studies, especially research concerning human-specific immune responses to infectious agents and drugs^28,30,31^.

Graft rejection is a severe disorder that has gained significant importance because of the increasing application of cell and tissue transplants^32^. It has been reported that the engraftment of immunologically incompatible mature cells into species such as rodent, avian, primate and human are capable of triggering graft rejection responses^32–36^. Graft rejection is the most frequent complication after transplantation and is a consequence of interactions between antigen-presenting cells of the recipients and mature T cells of the donor^37,38^. In clinics, mature T cells have to be depleted from donor tissues or only purified stem/progenitor cells can be used for transplantation in order to reduce the risk of rejection^39,40^. Because of this, the success of clinical transplantation is largely limited by the immunological incompatibility between donor and host cell/tissue and the high cost of tissue processing^32^. Additionally, in order to achieve successful and stable long-term reconstitution of human immune cells in humanized mice, purified stem cells completely devoid of mature T cells are required to prevent the development of graft rejection^41^.

In this study, we adopted the concept of humanized mouse models^24^ and aimed to stably reproduce bat’s biological system, particularly the immune system, in mice, by transplanting bat cells (*Eonycteris spelaea*) into immunodeficient mouse strain NOD-scid IL-2Rγ^−/−^ (NSG) mice (bat-mice). In this bat-mouse model, bat immune cells were successfully reconstituted in mice and unique features, such as resistance to graft rejection and functional demonstration of antigen-specific bat antibody responses have been observed and characterized.

## Results

### Transplantation of bat bone marrow (BM) cells led to stable reconstitution of bat immune cells in NSG recipients

**Figure 1:**
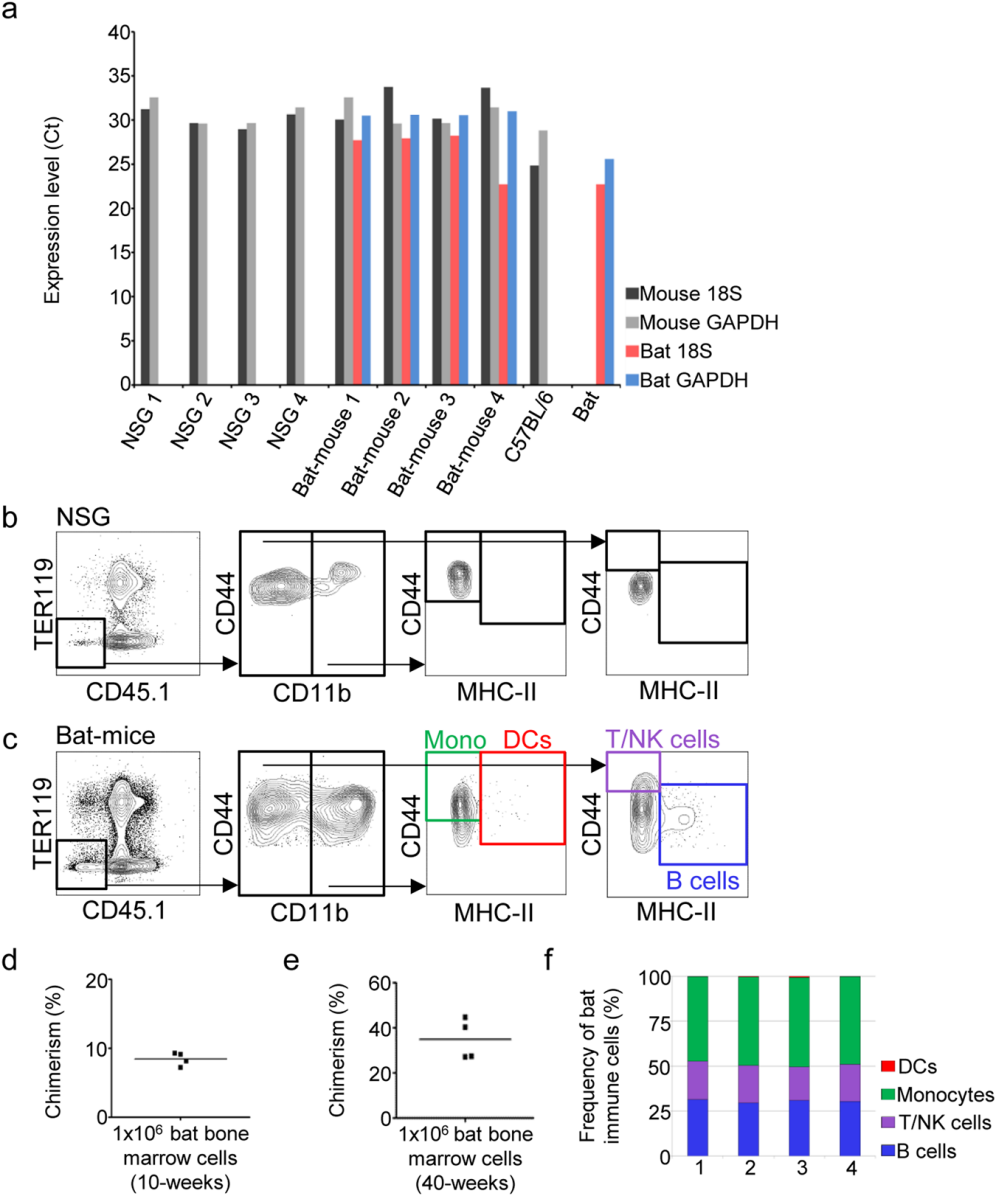
Measurement of reconstitution by qPCR and flow cytometry. Adult NSG mice were injected with 1 × 10^6^ bat bone marrow cells. (**a**) Detection of bat cells in bat-mice by species-specific primers. Peripheral blood samples were taken from NSG, C57BL/6, bat-mice 40-weeks post-injection, as well as bats, and assayed by qPCR with primers specific for mouse GAPDH, mouse 18S RNA, bat GAPDH and bat 18 S RNA. Data shown are the average cycle threshold (CT) values obtained from triplicates of each sample. (**b–c**) Forty weeks post-injection, peripheral blood from NSG (**b**) (*n* = 4) and bat-mice (**c**) (*n* = 4) were stained for CD45.1, Ter119, CD11b, CD44, MHC-II and analyzed by flow cytometry. Concatenated staining profiles are shown. (**d–e**) Chimerism levels in peripheral blood of adult NSG mice, ten weeks (**d**) and 40 weeks (**e**) post bat BM cell injection. Each symbol represents one mouse and the horizontal line indicates the mean value. (**f**) Proportions of various bat immune cell populations in bat-mice 40 weeks post-transplantation.

Due to their immunodeficiency, NSG mice are permissive for the engraftment of foreign cells^30^. To investigate their ability to support the engraftment of bat cells, we created bat-mice by transferring 1 × 10^6^ whole bat BM cells intravenously into eight-week old adult NSG mice. Ten weeks post-transplantation, blood samples were collected and analyzed for bat and mouse genes with species-specific primers (Supplementary Table 1) by quantitative polymerase chain reaction (qPCR). Untreated NSG mice were also kept in parallel as controls. Wild bat (*E. spelaea*) and C57BL/6 mice were used as both positive and negative controls, depending on the species-specific primers used. The results showed the presence of bat housekeeping genes (GAPDH and 18 S) within bat-mice, but not in control NSG mice (Fig. 1a). To further dissect the immune cell composition in blood, peripheral blood from both NSG and bat-mice were analyzed via flow cytometry. Due to the lack of bat-specific antibodies, antibodies such as anti-mouse CD11b, CD44, and major histocompatibility complex class II (MHC-II), which showed cross reactivity with bats previously^20,42^ and are able to specifically bind to *E. spelaea* cells (Supplementary Fig. 1) were used. As shown in Fig. 1b and c, mouse-specific CD45.1 and Ter119 antibodies were used to gate out majority of the mouse leukocytes and erythroid lineage cells. CD45.1^−^Ter119^−^ population was further separated into two populations by CD44 and CD11b antibodies. Within the CD44^+^CD11b^+^ population, monocytes and dendritic cells (DCs) were gated out by further staining with CD44 and MHC-II. From CD44^+^CD11b^−^ cell population, CD44 and MHC-II were used to distinguish between T/natural killer (NK) and B cells. All of the major bat immune cell populations, such as monocytes, T/NK cells, B cells and DCs were found within the peripheral blood of bat-mice (Fig. 1c). Bat chimerism in bat-mouse peripheral blood was calculated by analyzing the cells, which stained negative for both mouse CD45.1 and Ter119, using the formula: [%mCD45.1^−^Ter119^−^/(mCD45.1^−^Ter119^−^ + mCD45.1^+^Ter119^−^)]. At 10 weeks post-reconstitution, the percentage of bat chimerism ranged from ~7% to 9% in the peripheral blood of four separate bat-mice in the group (Fig. 1d). Forty weeks post-transplantation, an increase in reconstitution levels, ranging from ~20% to 40%, was observed, proving long-term reconstitution is viable in the bat-mouse model (Fig. 1e). Based on the profile of bat immune cells, monocytes made up the largest proportion of cells at ~45% to 50%, followed by B cells at ~20% to ~25%, T/NK cells at ~30% to 32% and DCs at ~0.2–1%, (Fig. 1f). Taken together, these results indicate that bat BM cells have a long-term repopulating capacity to survive, expand and stably establish a bat immune system in NSG recipients.

### Bat cells did not induce graft rejection in NSG

**Figure 2:**
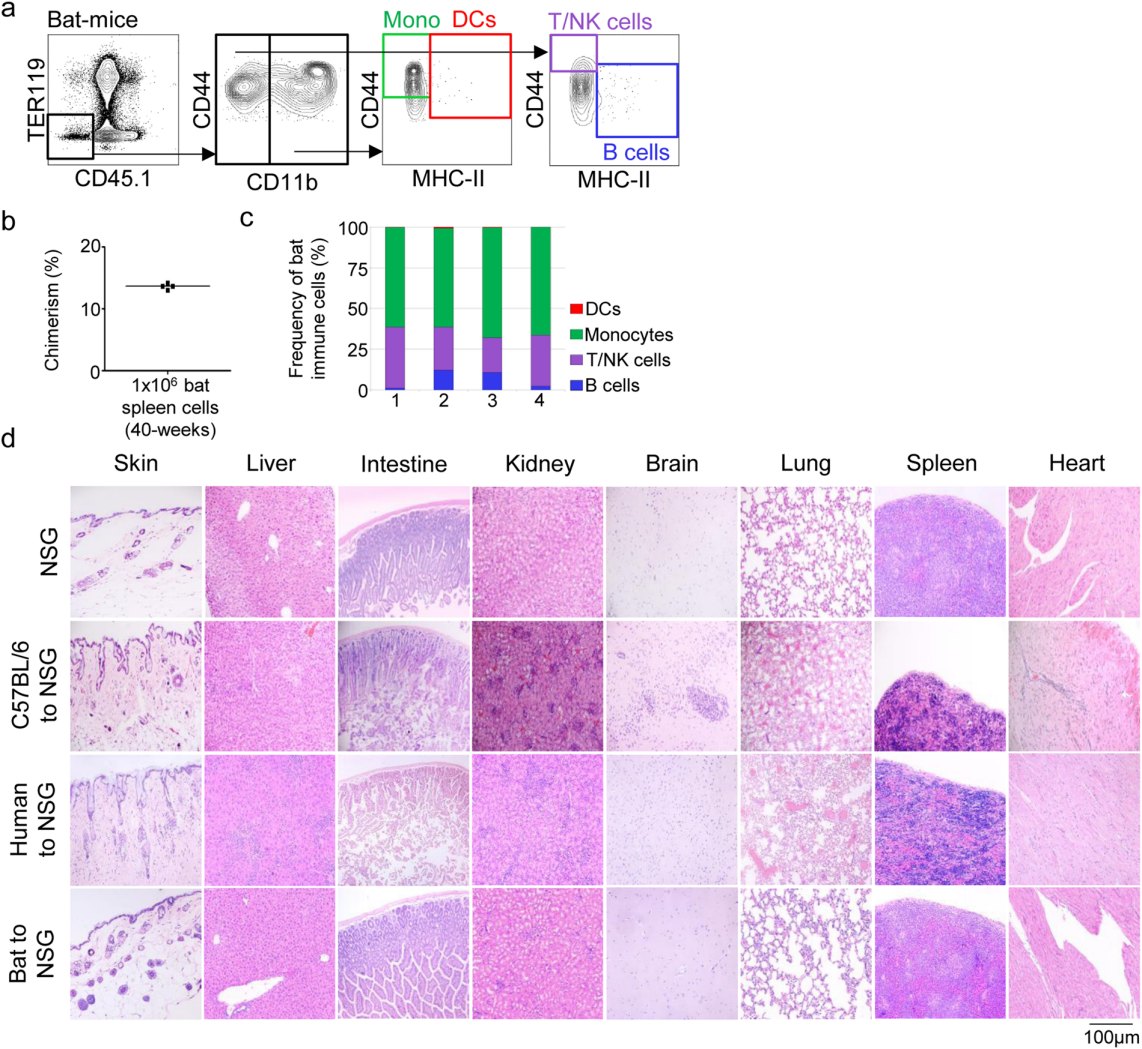
Assessment of potential graft rejection in bat-mice. (**a**) Adult NSG mice were injected with 1 × 10^6^ bat spleen cells per mouse. Peripheral blood from bat-mice (*n* = 4) were stained for CD45.1, Ter119, CD11b, CD44 and MHC-II and analyzed by flow cytometry. Concatenated staining profiles are shown. (**b**) Chimerism levels in peripheral blood of adult NSG mice injected with bat spleen cells, 40-weeks post-injection. Each symbol represents one mouse and the horizontal line indicates the mean value. (**c**) Proportions of various bat immune cell populations in bat-mice. (**d**) Histological analysis of organs from 40-weeks old NSG control mice (*n* = 4), NSG mice engrafted with 1 × 10^6^ C57BL/6 spleen cells 2-weeks post injection (*n* = 4), NSG mice engraft with 1 × 10^6^ human PBMCs 4 weeks post-injection (*n* = 4) and NSG mice engrafted with 1 × 10^6^ bat spleen cells 40 weeks post-injection (*n* = 4). Paraffin slides made from indicated organs were processed and stained with H&E. Representative images are shown. Scale bar applies to all panels.

Transplantation of an autologous or syngeneic graft will not trigger a rejection^32^. However, with a xenogeneic graft, where the donor and recipient are genetically different, the recipient will develop graft rejection^38^. The transfer of mature bat lymphoid cells was expected to cause the development of symptoms related to graft rejection. However, it was surprising to observe that the transplantation of bat cells did not develop any signs of graft rejection in bat-mice even 40 weeks after initial cell injection. To investigate if bat cells would generate a rejection response in NSG mice, bat splenocytes (1 × 10^6^) were used for transplantation, as majority of the cells within this organ are mature immune cells^38,43^. Forty weeks after injection, mice appeared healthy with no signs of graft rejection observed. Monocytes, T/NK cells, B cells and DCs were present in NSG mice engrafted with bat splenocytes (Fig. 2a). The reconstitution levels of bat immune cells in these mice ranged from ~12% to 15% (Fig. 2b). The overall proportion of bat monocytes, T/NK, B cells and DCs were ~60% to 65%, ~30% to 38%, ~5% to 10% and ~0–1% respectively in peripheral blood (Fig. 2c). The successful reconstitution with bat splenocytes suggested that bat mature immune cells might have the ability to expand and subsequently lead to long-term *in vivo* repopulation. This novel finding has never been observed before in species such as, humans, mice and other mammals, which had been tried in such studies^34–36,38,43–49^.

In order to uncover if bat-mice had immune-mediated tissue damage in the absence of observable clinical signs, histological analysis was applied to assess pathological changes in different organs from bat-mice. In most acute symptoms of graft rejection, the first organs affected with tissue damage are the liver, skin and intestinal tract^34,50^. Transplantation of C57BL/6 splenocytes and human peripheral mononuclear cells (PBMCs) separately into NSG mice were used as a positive control as they have been known to induce graft rejection in recipients with different genetic backgrounds^35,38,43,44^. Two to four weeks post-transplantation, recipients of 1 × 10^6^ C57BL/6 splenocytes or 1 × 10^6^ human PBMCs displayed symptoms of runt disease, a condition that features small and weaken mice, characteristic of graft rejection^51^. Organs were harvested from these mice and compared to bat-mice via histological and pathological analysis. Massive cell infiltration and damages were observed in various organs from mice that received C57BL/6 splenocytes or human PBMCs, while there was no significant difference between NSG mice and bat-mice, both of which were without any evident signs of clinical abnormalities (Fig. 2d). These results confirmed that mature bat immune cells did not induce rejection in NSG mice.

### Optimization of bat cell engraftment in NSG

**Figure 3:**
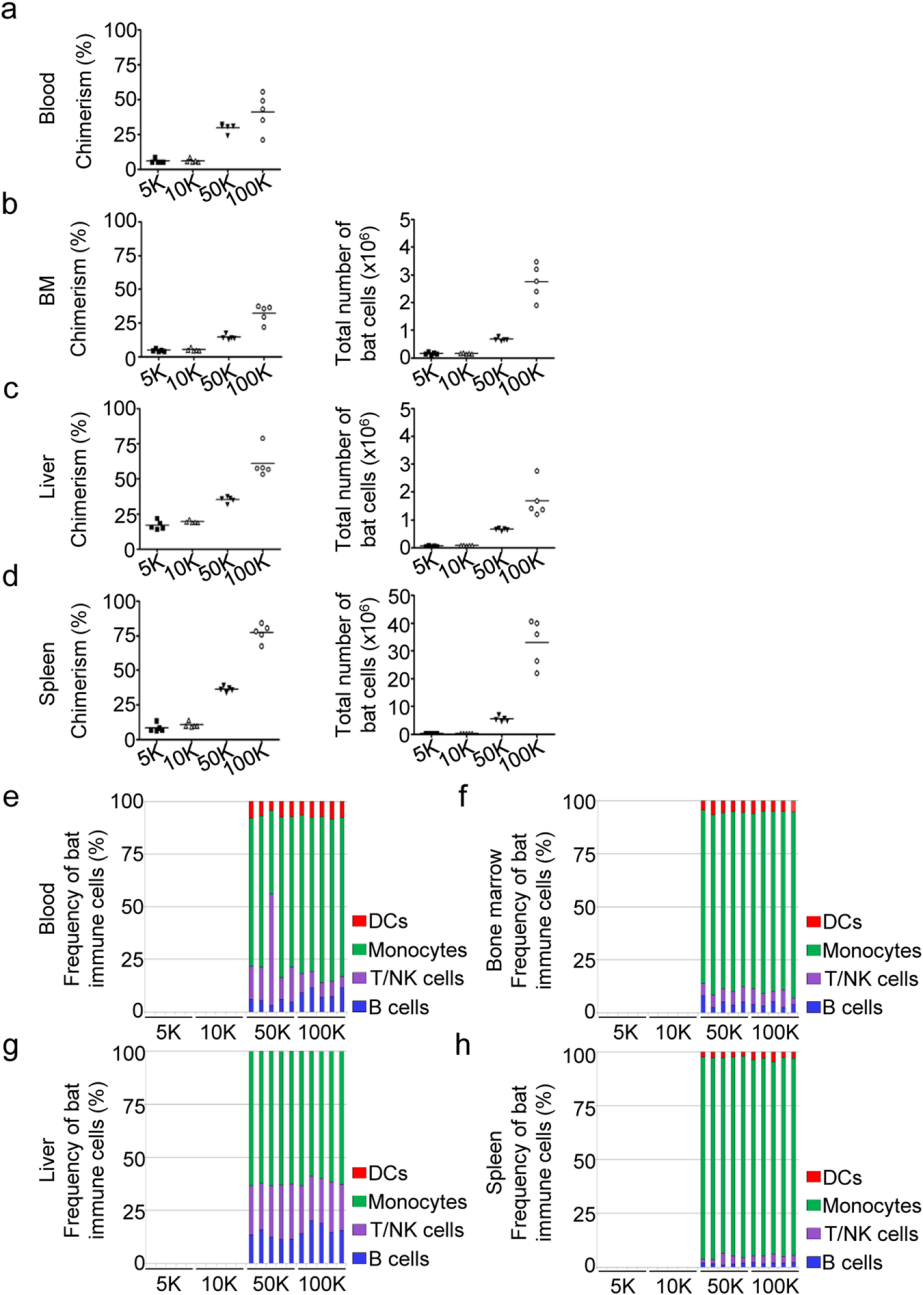
*In vivo* transplantation of bat bone marrow (BM) cells with limiting dilution. Bat BM cells were isolated and injected into sub-lethally irradiated NSG pups at 5 × 10^3^ (5 K), 1 × 10^4^ (10 K), 5 × 10^4^ (50 K) and 1 × 10^5^ (100 K) cells per mouse (*n* = 5 for each group). Forty-weeks later, the mice were cheek bled and blood was prepared and analyzed by flow cytometry. (**a**) Shown is the plot of chimerism levels within the peripheral blood of individual mouse injected with indicated number of bat bone marrow cells. Each symbol represents one mouse and the horizontal line indicates the mean value. (**b–d**) Chimerism levels and total bat cell count in the (**b**) bone marrow, (**c**) liver and (**d**) spleen of individual mouse injected with indicated number of bat bone marrow cells 40 weeks post-transplantation. Each symbol represents one mouse and the horizontal line indicates the mean value. (**e–h**) Percentage proportion phenotypic lymphocyte subset analysis within the (**e**) peripheral blood, (**f**) bone marrow, (**g**) liver and (**h**) spleen of bat-mice injected with bat bone marrow cells.

To optimize the production of bat-mice, further studies were conducted to improve the transplantation protocol and define the minimum number of bat BM cells needed to reconstitute NSG mice^52–54^. As compared to adult mice, neonatal NSG are known to be better at establishing human cell repopulation in humanized mice^25,55^. Bat BM cells were prepared and injected into sub-lethally irradiated NSG neonates at varying numbers of 5 × 10^3^, 1 × 10^4^, 5 × 10^4^ or 1 × 10^5^ cells. As shown in Fig. 3a, according to peripheral blood reconstitution levels at 40 weeks post-injection, the groups of 5 × 10^3^ and 1 × 10^4^ cells did not give rise to significant reconstitution, whereas a transfer of 5 × 10^4^ or more cells resulted in engraftment. However, to achieve a robust establishment and maintenance of bat immune cells in NSG recipients, an initiating number of 1 × 10^5^ bat BM cells was required to reach a reconstitution level of ~20% to 50% (Fig. 3a), which was comparable to adult NSG mice receiving 1 × 10^6^ BM cells (Fig. 1c). Systemic reconstitution in various organs such as BM, liver and spleen were analyzed (Supplementary Fig. 2). The bat cell chimerism levels in organs from groups of 5 × 10^3^, 1 × 10^4^ and 5 × 10^4^ cells remained low. In mice injected with 1 × 10^5^ cells, the reconstitution levels was robust with variation in different organs: in BM, levels of bat chimerism was ~20% to 35%, with bat cell numbers ranging from ~2 × 10^6^ to 3 × 10^6^ (Fig. 3b); in liver, reconstitution levels could reach ~50% to 75% with bat cell numbers of ~1 × 10^6^ to 2 × 10^6^ (Fig. 3c); spleen had the highest reconstitution levels and cell numbers which were ~70% to 80% and ~20 × 10^6^ to 40 × 10^6^ respectively (Fig. 3d). The dramatic increase in the number of bat cells within the organs compared to the initiating number of cells injected, demonstrated that there was a massive *in vivo* expansion of bat cells in NSG mice (Fig. 3b to d). Overall proportions of immune subsets, such as monocytes, T/NK, B cells and DCs also varied between the peripheral blood, bone marrow, liver and spleen. In the peripheral blood, monocyte levels were ~40% to 70%, T/NK cells ~20% to 58%, B cells ~2% to 5% and DCs ~2% to 5% (Fig. 3e). In the bone marrow, monocytes ~80% to 95%, T/NK cells ~2% to 5%, B cells ~2% to 5% and DCs ~2% to 5% (Fig. 3f). Within the liver, the proportion of monocytes was ~60% to 62%, T/NK cells ~20% to 30%, B cells ~10% to 20% and DCs ~0% to 0.05% (Fig. 3g). The spleen was mostly dominated by monocytes, ~90% to 95%, with T/NK, B cells and DCs standing at ~2% to 5%, ~1% to 3% and ~0.5% to 2% respectively (Fig. 3h). Altogether, it is evident that neonatal mice, with an engraftment of a limited number of bat BM cells, were able to achieve considerable levels of chimerism with all major immune cells present, therefore, enabling the reliable generation of a large cohort of bat-mice. Using the optimised protocol, we envisage that 80 to 100 bat-mice could potentially be generated from the BM of a single *E. spelaea* bat.

### The reconstituted bat immune system is functional in bat-mice

**Figure 4:**
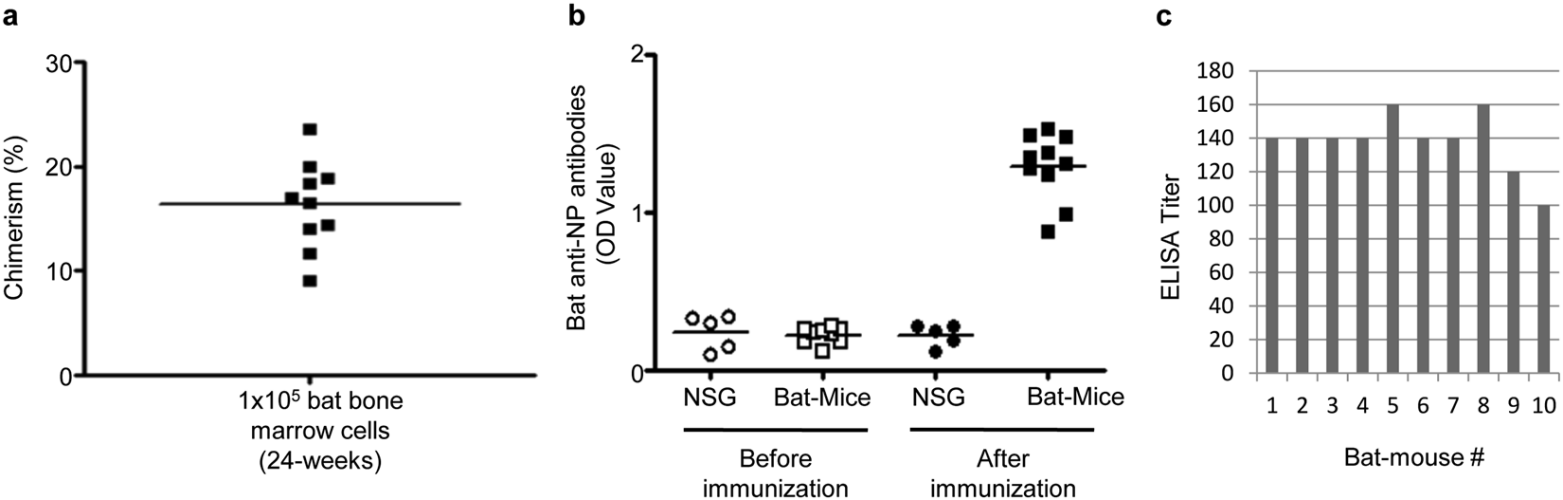
Determination of antigen-specific antibody response in bat-mice. Bat BM cells were isolated and injected into sub-lethally irradiated NSG pups at 1 × 10^5^ cells per mouse (*n* = 10). Twenty-four weeks post injection, the mice were cheek bled and blood was prepared and analyzed by flow cytometry. (**a**) Shown is the plot of chimerism levels within the peripheral blood of individual mouse before immunization. Each symbol represents one mouse and the horizontal line indicates the mean value. (**b**) Bat-mice (*n* = 10) and NSG mice (*n* = 5) were immunized via intraperitoneal injection with NP-KLH, using IFA as an adjuvant. Sera were collected from immunized bat-mice and NSG controls 2 weeks after the second booster. Optical density _450_ (OD_450_) levels of NP-specific antibodies were quantified by ELISA. Each symbol represents one mouse and the horizontal line indicates the mean value. (**c**) ELISA titres determined for each of the immunized bat-mice with the non-immunized bat-mice pre-bleed sera negative control. The titre was calculated from the reciprocal of the greatest dilution that yielded the ELISA reading ≥3 standard deviations above the negative control mean.

The demonstration of a functional bat immune system in NSG mice recipient holds a fundamental importance for the potential utility of this model. To investigate if bat-mice have a functional immune system, 24-week old bat-mice that were generated by injecting 1 × 10^5^ BM cells during their neonatal stage (Fig. 4a) were immunized with 4-Hydroxy-3-Nitrophenylacetyl hapten conjugated to keyhole limpet hemocyanin (NP-KLH) using incomplete Freund’s adjuvant (IFA) as an adjuvant to determine whether antigen-specific adaptive immune responses can be achieved in bat-mice. An ELISA based system was used to detect NP-specific bat antibodies using NP31-BSA as the ELISA antigen so that only NP-reacting antibodies will lead to a positive reading. Sera samples from the 10 immunized bat-mice showed positive reactivity with NP-antigen whereas the pre-bleed of each bat-mice and samples from the immunized NSG control mice were all negative (Fig. 4b). In addition, ELISA titers were determined for each of the immunized bat-mice (Fig. 4c). To confirm that antibodies produced in bat-mice were bat-specific, western and dot blot experiments utilizing anti-bat IgG antibody not only demonstrated the specificity of the goat anti-bat IgG antibody used in this study but also affirmed that only bat and bat-mice were capable of producing bat IgG antibodies. In bats and bat-mice, IgG was detectable at a dilution of 1:2000 and 1:1000, respectively (Supplementary Fig. 3). All together, these results confirmed that bat-mice were able to generate an antigen-specific response, therefore suggesting the presence of functional immune cells.

## Discussion

Bats are the only mammals capable of sustained flight and are increasingly recognized as one of the most important natural reservoir for some of the world’s most lethal viruses, including Nipah, Hendra, Ebola, Marburg and SARS-CoV. This, combined with their exceptional longevity and low tumorigenesis^11^, has raised an intriguing question on whether these seemingly unrelated biological features are actually intertwined and that they all represent different outcomes of the long evolutionary adaption to flight – an absolute unique biological ability only acquired by bats among all mammals. Comparative genomic studies have revealed an unexpected concentration of positively selected genes in the DNA damage checkpoints and immunity pathways that may be related to the origin of flight^56^. More recently, we have reported findings from a wider comparative genomic study, indicating bats are the only mammals which have lost the entire PYHIN gene family, which encodes DNA sensors highly important for dsDNA-triggered inflammation^57^. Taken together, these preliminary discoveries point to the possibility that bats have evolved an immune system, or an innate defense system that is more robust and tolerant to stress signals or stimuli (such as infection and DNA damage) as compared to other mammals, which in turn enables them to live longer and be more resilient to various pathogens.

While there is an urgent need to carry out in-depth bat research to unlock some of the mysteries associated with the above-stated observations and preliminary findings, bat research suffers major obstacles due to the lack of bat-specific research tools (such as antibodies and specific cell lines) and, more importantly, the lack of experimental animal system due to the outbreed nature and low reproduction rates of bats. To accelerate bat research in the immediate future, we propose to fill the gap using the bat-mouse platform to provide a genetically consistent model to carry out *in vivo* studies on bat immunity and for other biological research. In this study, we presented data to demonstrate that we not only generated a bat-mouse platform, but also showed that only 1 × 10^5^ bat BM cells are needed to generate one bat-mouse, i.e., approximately 80 to 100 bat-mice from one bat. This has solved two substantial hurdles for *in vivo* bat research: animal numbers and genetic homogeneity.

In addition to the successful reconstitution of bat immune cells in mice, this study also revealed an important, and unexpected, discovery that successful engraftment of mature immune cell xenograft can be achieved with no signs of clinical abnormalities. First of all, based on the results of engrafting mature splenocytes in mice, it is possible that mature immune cells in bats, such as monocytes, T/NK, B cells and DCs have some long-lived recirculating and expansion ability. However, we cannot rule out the possibility that reconstitution by rare recirculating stem cells or progenitors may occur. This interesting hypothesis is worth further investigation to examine possible new insights into unusual mechanisms for mature cell renewal and reprogramming in bats. Secondly, observed resistance to rejection is noteworthy and may suggest that transplantation of mature bat immune cells into hosts which are not necessarily genetically and MHC matched might not lead to rejection. In order to generate a graft rejection response, 3 key players need to be present, an antigen source, functional T cells and antigen presenting cells from the host. The lack of graft rejection symptoms can be attributed to multiple reasons, one of the main concern is can bat T cells recognise murine MHC molecules and other co-stimulatory molecules. Bat T cells could be primed to recognise the murine antigen via bat MHC molecules on bat antigen-presenting cells (APC), but the inability to recognise murine MHC molecules will prohibit the mounting of an immune response against host cells. Detailed characterisation of the bat major histocompatibility complex class I (MHC-I) molecules revealed unique peptide binding grooves not observed in any other mammals^58^. The hypothesized unique bat MHC-I molecule^58^ could potentially contribute to the development of a T cell receptor (TCR) with different molecular structure on bat CD8^+^ T cells during positive selection in thymus. Hence, the bat TCR could lack the ability to recognise murine MHC-I molecules and thus, develop a compromised cytotoxic T lymphocytes response. However, these are speculations that require further investigation, which is not within the scope of this pilot, proof-of-concept study. The new model serves as a useful tool to explore deeper mechanisms on how bat immune cells develop resistance to graft rejection in the bat-mouse model while maintaining their functionality. This research would not only benefit the study of graft rejection, but also aid in the development of drugs used to curtail undesirable effects of transplantations.

The normal regulatory interactions that lead to homeostasis within an intact immune system, appears to be unaltered in bat BM cell-reconstituted NSG mice. Even though the study of immune cell responses in bat-mice is restricted due to the current shortage of bat-specific research tools, the observation that a rapid development of bat-specific antibodies post NP-KLH immunization suggests that bat humoral immune responses involving antigen presenting cells and B cells within a mouse environment are intact and functional. Additionally, as KLH is known to be a T cell dependent antigen which primes antigen-specific T cell responses^59,60^, the responses to NP-KLH suggests that bat-mice could have developed antigen-specific T cell responses. Our novel bat-mouse model paves a new direction in the field of bat research, providing a powerful platform for dissecting the unique immune system and investigation of other bat biological processes.

Unlike the optimised humanized mouse model, our bat-mice were created with crude whole bone marrow cells without any hematopoietic stem cell modification^29,55^. Further optimisation is required to reveal the full potential of this novel model. An important point to note is that all phenotypic characterisation of bat immune cells derived from our bat-mice thus far were solely based upon the limited bat-reactive antibodies we possess. Though a larger panel of reagents had recently been established^42^, they were optimised for *Pteropus alecto* with limited cross-reactivity against *E. spelaea*. Various immune cells, especially T/NK cells, identified in our bat-mice to date will be further investigated when more reagents are available. More research is currently in progress to establish an *E. spelaea* cross-reactive panel to better characterise its immune cell profile, and subsequently help address our above-mentioned limitations.

In conclusion, while we are fully aware that bat research is only at its infancy, we are still facing an uphill battle to generate bat-specific research tools, especially antibodies. The novel bat-mouse model generated in this study represents a major technical advancement in recent years and we are confident that this will accelerate many aspects of bat research internationally in the near future. Moreover, the additional discovery that bat BM cells can be successfully engrafted without graft rejection is highly significant on two accounts. One, this unexpected discovery may represent one of the strongest evidence obtained to date in support of the notion that there is immune tolerance or a dampened innate defence system in bats. Two, this unique phenomenon, which is not typically seen in mainstream immunological studies of other mammals may open a new area for the study of immunity in unique species and create new opportunities to address human health issues. Together, this would not only allow a better understanding of bat immune responses to diseases and vaccines, but also for testing immunomodulators and exploring mechanisms in infection and neoplasia.

## Methods

### Mice

NOD-scid IL2Rγ ^−/−^ (NSG) mice (Stock #005557) were obtained from Jackson Laboratory and bred in the animal facility at A*STAR, Biological Resource Centre. Neonatal mice (100 rads), within 72 hours of birth, and 8-week old adult mice (250 rads) were sub-lethally irradiated and injected with bat cells via intra-hepatic^61^ and intra-venous injection, respectively. The International Animal Care and Use Committee (IACUC), A*STAR approved this study and assigned a protocol number (BRC #151039). All animal experimental procedures carried out were in accordance to the protocol’s guidelines and regulations.

### Bats

*Eonycteris spelaea* (common name, cave nectar bat), our species of interest, was captured in Singapore at dusk using mist nets and transferred to clean customized bat bags for transportation. All animal processing work was conducted in accordance to with approved guidelines, methods and permits from Duke-NUS Medical School and SingHealth Experimental Medicine Centre (2015/SHS/1088). Bats were anaesthetised using isoflurane and exsanguinated via cardiac bleed. Various samples, such as, spleen and bone marrow were harvested. The spleen tissues were mashed through a 100 µm filter (Thermo Fisher scientific, USA) in DMEM medium (Thermo Fisher scientific, USA). The single-cell suspension was washed and re-suspended in media supplemented with 10% fetal bovine serum (FBS). Bone marrows were processed by flushing femurs and tibias with 5 mL of DMEM medium using 5 mL syringes (BD Biosciences, USA) and 27 gauge needles (BD Biosciences, USA). Contents flushed out of the bone marrow were mashed through a 100 µm filter in DMEM medium. The single-cell suspension was washed and re-suspended in media supplemented with 10% FBS. Cell viability was assessed using trypan blue.

### Flow cytometry

Conjugated antibodies for mouse CD11b (M1/70; BD Biosciences, USA), MHC-II (2G9; BD Biosciences, USA), CD45.1 (A20; Biolegend, USA), Ter119 (TER-119; Biolegend, USA), CD44 (IML; eBioscience, USA) were used in flow cytometry assays. Cells were stained with antibodies, in 100 μl phosphate buffered saline (PBS) containing 0.2% BSA and 0.05% sodium azide for 30 minutes on ice. Flow cytometry was performed on an LSRII flow cytometer using the FACSDiva software (BD, USA); 1 × 10^4^ events were collected per sample, and analyzed using the Flowjo software version 10 (Treestar, Ashland, USA). Percentage chimerism was calculated using the formula, [%mCD45.1^−^Ter119^−^/(mCD45.1^−^Ter119^−^ + mCD45.1^+^Ter119^−^)].

### RNA isolation and quantitative polymerase chain reaction (qPCR)

Blood samples were collected from NSG, C57BL/6, bat-mice and bats. RNA was prepared from these samples using RNeasy Micro kit (Qiagen, Netherlands). Reverse transcription was performed using iScript cDNA Synthesis Kit (BIO-RAD, USA) according to manufacturer’s specifications. qPCR was subsequently performed in triplicates using SensiFAST™ SYBR No-ROX Kit (Bioline, USA) and assays were run on the CFX96 Touch™ Real-Time PCR Detection System (BIO-RAD, USA) under the following cycling condition: 95 °C for 5 minutes, followed by 40 cycles of 95 °C for 5 seconds and 58 °C for 30 seconds, and ending with a melt profile analysis. Bat- and mouse-specific qPCR primers were designed by first aligning the sequences from the two species, and subsequently targeting regions that are more than 50% (nucleotide) difference between the two species. Sequences of bat and mouse specific primers are listed in Supplementary Table 1.

### Immunization

Mice were immunized by injecting NP-KLH (Biosearch Technologies, USA) emulsified in IFA (Sigma-Aldrich, USA), intraperitoneally. To prepare emulsification, NP-KLH and IFA were added together and sonicated. One injection of 100 µg of NP-KLH was used to immunize the mice. After the first injection, mice were supplemented with 2 booster shots which were injected on the same day for 2 consecutive weeks. Mice were bled before immunization and bled again 2-weeks following the third injection for the detection of NP-specific antibodies.

### ELISA

Sera were collected before and after challenging with NP-KLH by cheek bleed. Bat immunoglobulin (Ig) content specific to NP-KLH antigens was assessed by ELISA. Briefly, microtiter plates (Thermo Fisher Scientific, USA) were coated with NP31-BSA (Biosearch Technologies, USA) at 10 µg/mL. Bat specific antibodies were detected with bat IgG heavy and light chain antibody (A140-118B; Bethyl Laboratories, USA), donkey anti-goat IgG-HRP (SC-2056; Santa Cruz Biotechnology, USA) and 3,3′,5,5′-tetramethylbenzidine (TMB) (Thermo Fisher Scientific, USA). Optical density (OD) was read at 450 nm with a plate reader (Tecan, USA).

### Western blot

Specificity of bat immunoglobulin G (IgG) was determined using western blot. Pooled sera from two individual bats (*E. spelaea*) and sera from C57BL/6 mice, both diluted 1:50 in PBS, were ran on a 12% SDS-PAGE gel, followed by transfer onto Polyvinylidene difluoride (PVDF) transfer membranes (Millipore, USA). Membranes were blocked in Tris-buffered saline with 0.1% Tween (TBS-T 0.1%) and 2.5% skim milk for 1 hour at room temperature (RT), followed by labelling with goat anti-bat IgG heavy and light chain antibody (A140-118B; Bethyl Laboratories, USA) diluted 1:1,000 in TBS-T 0.1% overnight at 4 °C. Membrane was washed 3 times for 5 minutes with TBS-T 0.1%, then labelled with donkey anti-goat IgG-HRP (SC-2056; Santa Cruz Biotechnology, USA) diluted 1:10,000 in TBS-T 0.1% at RT for 1 hour, and subsequently undergo a final 3 times 5 minutes wash. Membrane was visualized using enhanced chemiluminescence (ECL) prime chemi-luminescence reagent (GE Healthcare, USA) and a myECL Imager (Thermo Fisher Scientific, USA).

### Dot blot

Bat-specific IgG was analysed via dot blot. Bat-mice were cheek bled post NP-KLH immunizations, sera samples were dotted onto nitrocellulose membrane (Pore size 0.45 µm; BIO-RAD, USA) and left to dry for 30 minutes. Blocking buffer (Tris-buffered saline with polysorbate 20 (TBS-T) and 5% low-fat milk powder) was used to block non-specific sites for 30 minutes at RT with agitation. After washing, membrane was incubated for 1 hour at RT with goat anti-bat IgG heavy and light chain antibody (A140-118B; Bethyl Laboratories, USA) in PBS-T containing 2.5% milk and for 1 hour with agitation, followed by 3 times 5 minutes wash with TBS-T. Secondary antibody incubation was done with donkey anti-goat IgG-HRP (SC-2056; Santa Cruz Biotechnology, USA) in PBS-T containing 2.5% milk for 1 hour at RT with agitation and subsequently washed with TBS-T for 3 times 5 minutes before reaction development using enhanced ECL reagent (Thermo Fisher Scientific, USA). Results were developed in the dark room for 10 seconds with CL-XPosure^TM^ Film (Thermo Fisher Scientific, USA).

### Cell proliferation assay

Mesenteric lymph nodes from bat-mice were harvested, processed into single cell suspension and incubated with Cell Trace Violet (Invitrogen, USA) (0.5 μM final concentration) for 20 minutes at 37 °C with 5% CO_2_. To quench the reaction, 10% FBS was added to the cells and incubated for 5 minutes on ice. The cells were washed twice in PBS supplemented with 2% FBS and resuspended in complete medium, seeded at 1 × 10^5^ cells per well in a 96-well flat bottom plate and cultured for 5 days at 37 °C, 5% CO_2_, with either 2 μg/ml concanavalin A (ConA) (Sigma), KLH (Biosearch Technologies, USA) or media alone. After 5 days incubation, cells were stained with specific antibodies and analyzed by flow cytometry (as described above).

### Histology

Mouse organs were collected, fixed with 10% formalin and embedded in paraffin for processing into sections. Formalin-fixed paraffin sections (4–6 μm) were dewaxed by melting for 30 minutes at 65 °C, cleared in xylene twice for 5 minutes, and rehydrated in water-ethanol solutions containing decreasing percentages of ethanol. To determine tissue morphology, sections were stained with hematoxylin–eosin (Gill 2 Hematoxylin and Eosin Y alcoholic; Thermo Sandon, Cheshire, UK) following a standard procedure. Sections were imaged and analysed under an Olympus BX-61 microscope (Olympus, Japan).

### Statistical analysis

All data are reported and represented as mean.

### Data availability

All data generated or analyzed during this study are included in this published article (and its Supplementary Information files).

## Electronic supplementary material

Supplementary Material
