# Supplementary material for: Bat-mouse bone marrow chimera: a novel animal model for dissecting the uniqueness of the bat immune system
Source: Sci Rep. 2018 Mar 16;8:4726. doi: 10.1038/s41598-018-22899-1 (PMC5856848; doi:10.1038/s41598-018-22899-1)
Supplement: Supplementary file 1 — Supplementary Material [file 41598_2018_22899_MOESM1_ESM.docx]

**Bat-mouse bone marrow chimera: a novel animal model for dissecting the uniqueness of the bat immune system**

Kylie Su Mei Yong^1,*^, Justin Han Jia Ng^2,*^, Zhisheng Her^1^, Ying Ying Hey^2^, Sue Yee Tan^1^, Wilson Wei Sheng Tan^1^, Sergio Erdal Irac^2^, Min Liu^1^, Xue Ying Chan^1^, Merry Gunawan^1^, Randy Jee Hiang Foo^2^, Dolyce Hong Wen Low^2^, Ian Hewitt Mendenhall^2^, Yok Teng Chionh^2^, Charles-Antoine Dutertre^2,3^, Qingfeng Chen^1,4, 5,#^, Lin-Fa Wang^2,#^

**Supplementary Information**

| Genes | Sense | Antisense |
| --- | --- | --- |
| P.a. 18S | TACTGCAATTAAGGGTGTAGG | CATAATGGTGATTACACGTTC |
| M.m. 18S | ACTGCCATTAAGGGCGTGG | CATGATGGTGATCACTCGC |
| P.a. GAPDH | ACCAGGGCTGCTTTTAACTC | AACTTGCCATGGGTGGAATC |
| M.m. GAPDH | CAGGGCTGCCATTTGCAG | TGAATTTGCCGTGAGTGGAG |

**Supplementary Table 1.** **Primers for qPCR**. Primer sequences for bat and mouse genes.

P.a. – *Pteropus alecto*; M.m. – *Mus musculus*

**Supplementary Figure 1. Measurement of reconstitution within the blood and organs of *Eonycteris spelaea* via flow cytometry.** *E. spelaea* was sacrificed; blood and organs such as spleen and bone marrow (BM) cells were isolated, prepared, stained for CD11b, CD44, MHC-II and subsequently analyzed by flow cytometry.


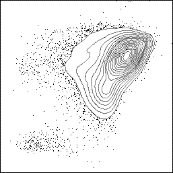

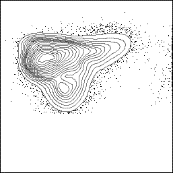

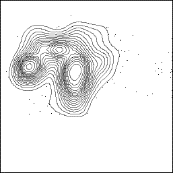

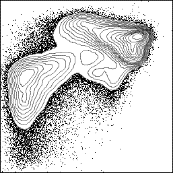

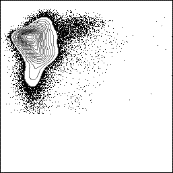

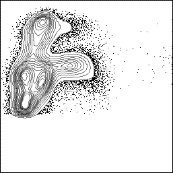

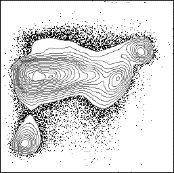

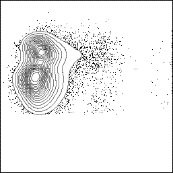

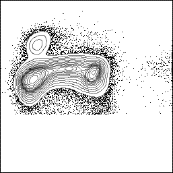


CD44

Blood

CD11b

MHC-II

MHC-II

CD44

CD44

CD44

Spleen

CD11b

MHC-II

MHC-II

CD44

CD44

CD44

Bone marrow

CD11b

MHC-II

MHC-II

CD44

CD44

Mono

DCs

Mono

DCs

Mono

DCs

T/NK cells

B cells

T/NK cells

B cells

T/NK cells

B cells

**Supplementary Figure 2. Measurement of reconstitution in organs from bat-mice by flow cytometry.** Bat bone marrow (BM) cells were isolated and injected into sub-lethally irradiated NSG pups at 1x10^5^ (100K) cells per mouse (*n* = 5). Forty weeks later, the bat-mice were sacrificed. Organs such as spleen, BM and liver were prepared, stained for CD45.1, Ter119, CD11b, CD44, and MHC-II and subsequently analyzed by flow cytometry. Concatenated staining profiles are shown.


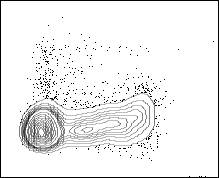


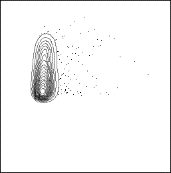

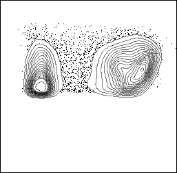

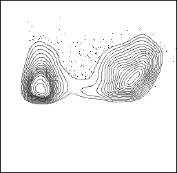

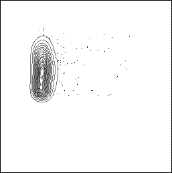

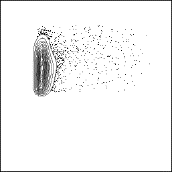

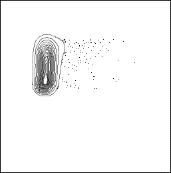

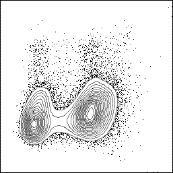


CD45.1

CD44

CD44

CD44

CD11b

MHC-II

MHC-II

TER119

CD44

CD44

CD44

TER119


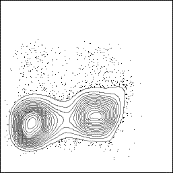

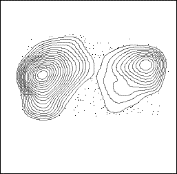

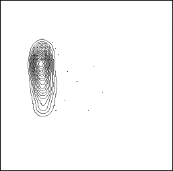

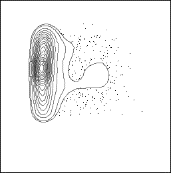


CD44

CD44

CD44

TER119

Spleen

Bone marrow

Liver

Mono

DCs

T/NK cells

B cells

Mono

DCs

T/NK cells

B cells

Mono

DCs

T/NK cells

B cells

CD45.1

CD11b

MHC-II

MHC-II

CD45.1

CD11b

MHC-II

MHC-II

**Supplementary Figure 3. Analysis of bat-specific antibody specificity and response in bat-mice.** (**a**) Pooled bat sera from 2 individuals (*E. spelaea*) and sera from a C57BL/6 mouse were ran on western blot to determine specificity of bat-specific antibody used. Presence of band on the membrane indicates presence of bat IgG binding. (**b**) Bat-mice (*n* = 10) were immunized via intraperitoneal injection with NP-KLH, using IFA as an adjuvant. Sera were collected from bat-mice 2 weeks after the second booster. Shown is the dot blot of representative samples from bat, bat-mice, NSG, BALB/c, C57BL/6 and phosphate buffered saline (PBS) which was used a diluent. Dot on the membrane represents the presence of bat IgG.

a


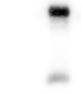


IgG Heavy Chain

(50 kDa)

IgG Light Chain

(25 kDa)

Pooled

*E.s.* sera

C57BL/6

Mouse sera

b


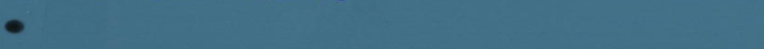

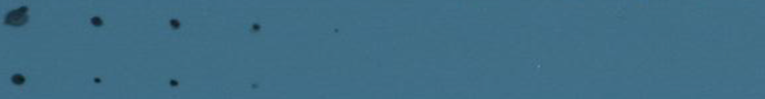

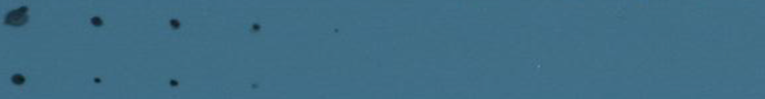


Bat

NSG

BALB/c

C57BL/6

PBS

Bat

Bat-mice

Controls

Neat

1:10

1:100

1:1K

1:2K

1:4K

1:8K

1:16K

1:32K

1:64K
